# Measurement of the Quantum Tunneling Gap in a Dysprosocenium Single-Molecule Magnet

**DOI:** 10.1021/acs.jpclett.3c00034

**Published:** 2023-02-22

**Authors:** William J. A. Blackmore, Andrea Mattioni, Sophie C. Corner, Peter Evans, Gemma K. Gransbury, David P. Mills, Nicholas F. Chilton

**Affiliations:** Department of Chemistry, School of Natural Sciences, University of Manchester, Oxford Road, Manchester M13 9PL, U.K.

## Abstract

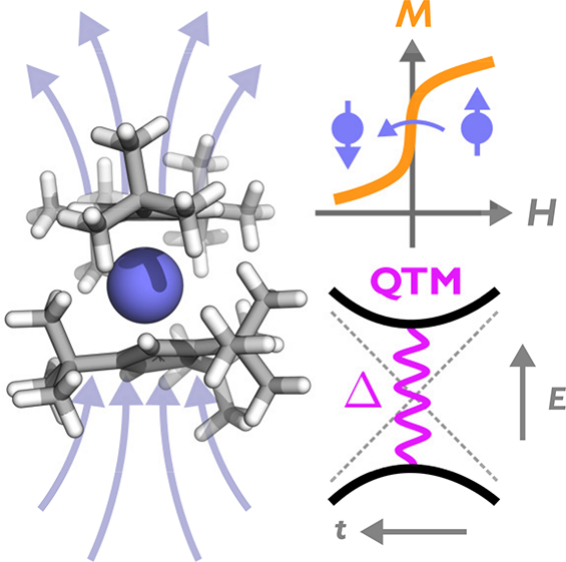

We perform magnetization sweeps on the high-performing single-molecule magnet [Dy(Cp^ttt^)_2_][B(C_6_F_5_)_4_] (Cp^ttt^ = C_5_H_2_^*t*^Bu_3_-1,2,4; ^*t*^Bu = C(CH_3_)_3_) to determine the quantum tunneling gap of the ground-state avoided crossing at zero-field, finding a value on the order of 10^–7^ cm^–1^. In addition to the pure crystalline material, we also measure the tunnel splitting of [Dy(Cp^ttt^)_2_][B(C_6_F_5_)_4_] dissolved in dichloromethane (DCM) and 1,2-difluorobenzene (DFB). We find that concentrations of 200 or 100 mM [Dy(Cp^ttt^)_2_][B(C_6_F_5_)_4_] in these solvents increases the size of the tunneling gap compared to the pure sample, despite a similarity in the strength of the dipolar fields, indicating that either a structural or vibrational change due to the environment increases quantum tunneling rates.

Single-molecule magnets (SMMs) are highly anisotropic paramagnetic molecules that have large energy barriers to reversal of their magnetic moment, leading to slow magnetic reversal. At cryogenic temperatures, the scarcity of phonons means that magnetization reversal is sufficiently slow such that the moment is often considered blocked on the time scale of the measurements, and hence can show memory effects.^1^ Rapid advancements in synthetic organometallic chemistry have recently led to vast increases in the magnitude of this barrier, raising the temperature at which memory effects can be observed.^2−5^

While the temperature at which magnetic hysteresis can be observed has increased with these high-performance organometallic SMMs, monometallic SMMs are still plagued by fast reversal at zero magnetic field which appears as a step in magnetic hysteresis loops. This fast reversal is commonly ascribed to quantum tunneling of the magnetization (QTM),^6^ which allows the magnetization to reverse under the energy barrier when the two ground states of the SMM come into resonance. QTM, where spins tunnel from state |*m*⟩ to |*m*′⟩, occurs due to the presence of an avoided crossing at zero external magnetic field (Figure 1). While the two states of a Kramers doublet |*m*⟩ and |*m*′⟩ are orthogonal projections of the total angular momentum, and therefore should always cross in zero external magnetic field, they are usually mixed by a small transverse magnetic field (e.g., dipolar or hyperfine field) causing Δ_*m*,*m*′_ ≠ 0 in the effective spin-1/2 Hamiltonian:
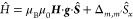
1where  are the effective spin-1/2 spin operators and ***g*** is the *g*-matrix of the ground state doublet. For non-Kramers ions, either a transverse magnetic field and/or the crystal field potential can open a tunnel splitting. In any case, slow (adiabatic) passage through the avoided crossing leads to magnetization reversal, and thus QTM can be observed experimentally via sharp steps in the magnetization as the field is swept through zero.^7−9^ At higher sweep rates, the spin has a nonzero probability to undergo diabatic passage and retain its magnetization; this is commonly called a Landau–Zener transition.^10,11^ For SMMs with small separations to excited states, different states on either side of the barrier can align at nonzero magnetic fields, and it is not uncommon for numerous steps to be observed within experimentally accessible magnetic fields.^12−17^

**Figure 1:**
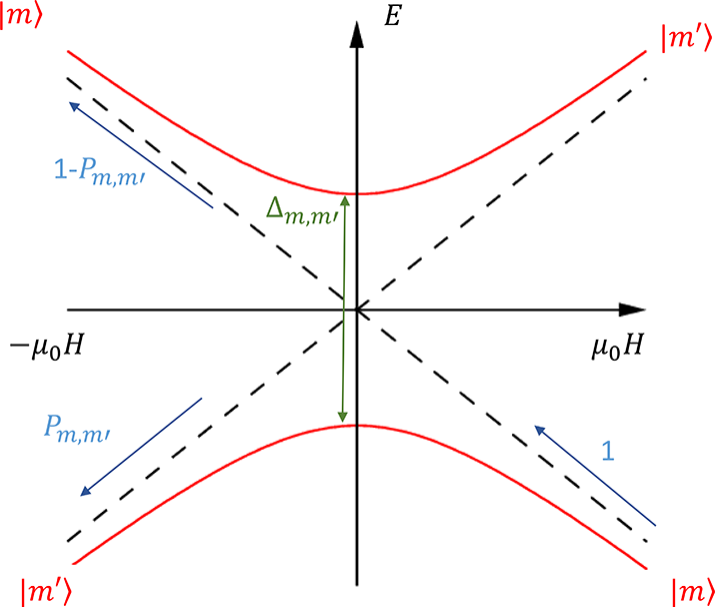
QTM and Landau–Zener transitions. Red curves indicate the eigenstates of eq 1, while black dashed curves are the eigenstates when Δ_*m*,*m*′_ = 0. When a molecule is subject to a large positive field at very low temperatures, it is in equilibrium in state |*m*⟩ (bottom right) and the sample is magnetized. When the field is swept toward negative values slowly, the molecule adiabatically follows the red curve and ends up in state |*m*′⟩ (bottom left). If the field is swept quickly, there is a nonzero probability 1 – *P*_*m*,*m*′_ that the molecule makes a diabatic transition and remains in |*m*⟩.

Theoretically, QTM should be largely unaffected by any changes in the surroundings of the magnetic ion,^18,19^ as observed experimentally for Mn_12_ and Fe_8_.^20^ However, recently we observed that the zero-field step in hysteresis loops for [Dy(Cp^ttt^)_2_][B(C_6_F_5_)_4_] (Cp^ttt^ = C_5_H_2_^*t*^Bu_3_-1,2,4; ^*t*^Bu = C(CH_3_)_3_) changed in dichloromethane (DCM) and 1,2-difluorobenzene (DFB) solutions, in comparison to the solid crystalline phase, with a counterintuitive concentration dependence.^2^ This suggests that the local environment of the [Dy(Cp^ttt^)_2_][B(C_6_F_5_)_4_] molecule does have an effect on the zero-field QTM properties, which we attributed to either a relaxation of the local geometry or availability of different phonon modes. To directly quantify the tunnel splitting in [Dy(Cp^ttt^)_2_][B(C_6_F_5_)_4_] and investigate further, here we measure sweep-rate-dependent demagnetization curves of a pure crystalline sample of [Dy(Cp^ttt^)_2_][B(C_6_F_5_)_4_] and frozen solutions in DFB and DCM with various concentrations. From these experiments we extract the size of the QTM tunneling gaps using the Landau–Zener protocol and compare the effect of environment and concentration. We find that concentrations of 200 and 100 mM in both solvents increases the size of the tunneling gap by ∼2 times compared to the pure sample, despite the average Dy···Dy distance increasing, and hence conclude that structural and/or vibrational changes increase QTM in the frozen solution phase. As the concentration of [Dy(Cp^ttt^)_2_][B(C_6_F_5_)_4_] is decreased to 10 mM, we find the size of the tunneling gap becomes more comparable with the pure sample.

To determine the transition probability across a tunneling gap Δ at a constant sweep rate d*H*/d*t*, we use the equation defined by the Landau–Zener–Stuckelberg (LZS) model:
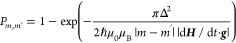
2where |*m* – *m*′| is the change in the angular momentum projection upon tunneling (Figure 1) and ***g*** is the *g*-matrix.^7,8,21^ There is an amendment to the LZS calculated by Kyanmuma, Garg, and Vijayaraghavan to account for energy fluctuations and incoherent transitions (eq S1).^22^ However, in the analysis of our field sweeps, we find little difference between the two models (Figures S1 and S2) such that both models give the same conclusions. We therefore focus on the results obtained with the LZS model.

The field sweeps were performed at 1.8 and 4 K, and therefore, only the ground Kramers doublet is relevant and [Dy(Cp^ttt^)_2_][B(C_6_F_5_)_4_] can be modeled using eq 1. In the effective spin-1/2 model, the value of |*m* – *m*′| = 1 and for [Dy(Cp^ttt^)_2_][B(C_6_F_5_)_4_] ***g*** = diag(0, 0, 19.98).^23^ As our measurements are performed on polycrystalline or solution samples, we must integrate the magnetic field over all orientations. Due to the very strong eas*y*-axis anisotropy of the ground doublet of [Dy(Cp^ttt^)_2_][B(C_6_F_5_)_4_], this can be obtained analytically by integrating eq 2 between the hard-plane and the eas*y*-axis:
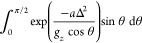
3where
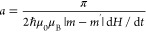
4

Performing this calculation, we find that the powder-averaged probability is
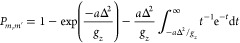
5where
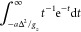
6is the incomplete gamma function .

For each field sweep, linear fits either side of the step at zero-field were performed to obtain the magnetization value before and after the QTM transition. The range of these fits was over linear regions of the data determined by eye (Figure 2; the ranges have been explored to obtain estimated uncertainties) and are used to calculate *P*_*m*,*m*′_ via^7^
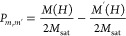
7where *M*(*H*) and *M*′(*H*) are the calculated *y*-intercept values before (high *M*) and after (low *M*) the transition, respectively. *M*_sat_ is the magnitude of the magnetization at +2 T. The values of *P*_*m*,*m*′_ calculated in eq 7 are then used to determine the value of Δ in eq 5. Errors were estimated by increasing the range of the fit by 800 Oe at both ends and comparing the difference in Δ to the initial model. The value 800 Oe is equivalent to one data point in a 200 Oe/s field sweep (see Figure 2).

**Figure 2:**
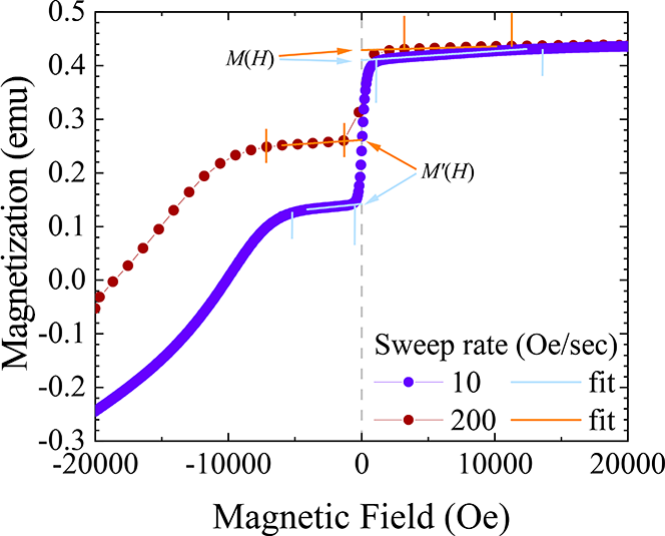
Visual representation of the fitting method. Linear regions are determined by eye and fitted to *y* = *mx* + *c* as shown by the approximately horizontal lines. *M*(*H*) and *M*^′^(*H*) in eq 7 are determined from the *c* values in the linear fit. The fit was increased by ≈800 Oe on either side, the range of which is visualized by the vertical lines, to determine the error Δ.

Field sweeps from +2 T → – 2 T are shown in Figures 3 and 4. For the pure [Dy(Cp^ttt^)_2_][B(C_6_F_5_)_4_] sample secured in eicosane, the 10 Oe/s demagnetization data show a slow linear decrease from +2 T before a sharp step at zero field (Figure 3a). After the transition there is a slow linear decrease before a broad downturn at −1 T. At faster sweep rates, the magnitude of the QTM step at zero field decreases, and the downturn below −1 T becomes broader and shifts to more negative fields. Changing the temperature from 1.8 to 4 K appears to have little effect on the magnetization over the whole range (Figure 3b).

**Figure 3:**
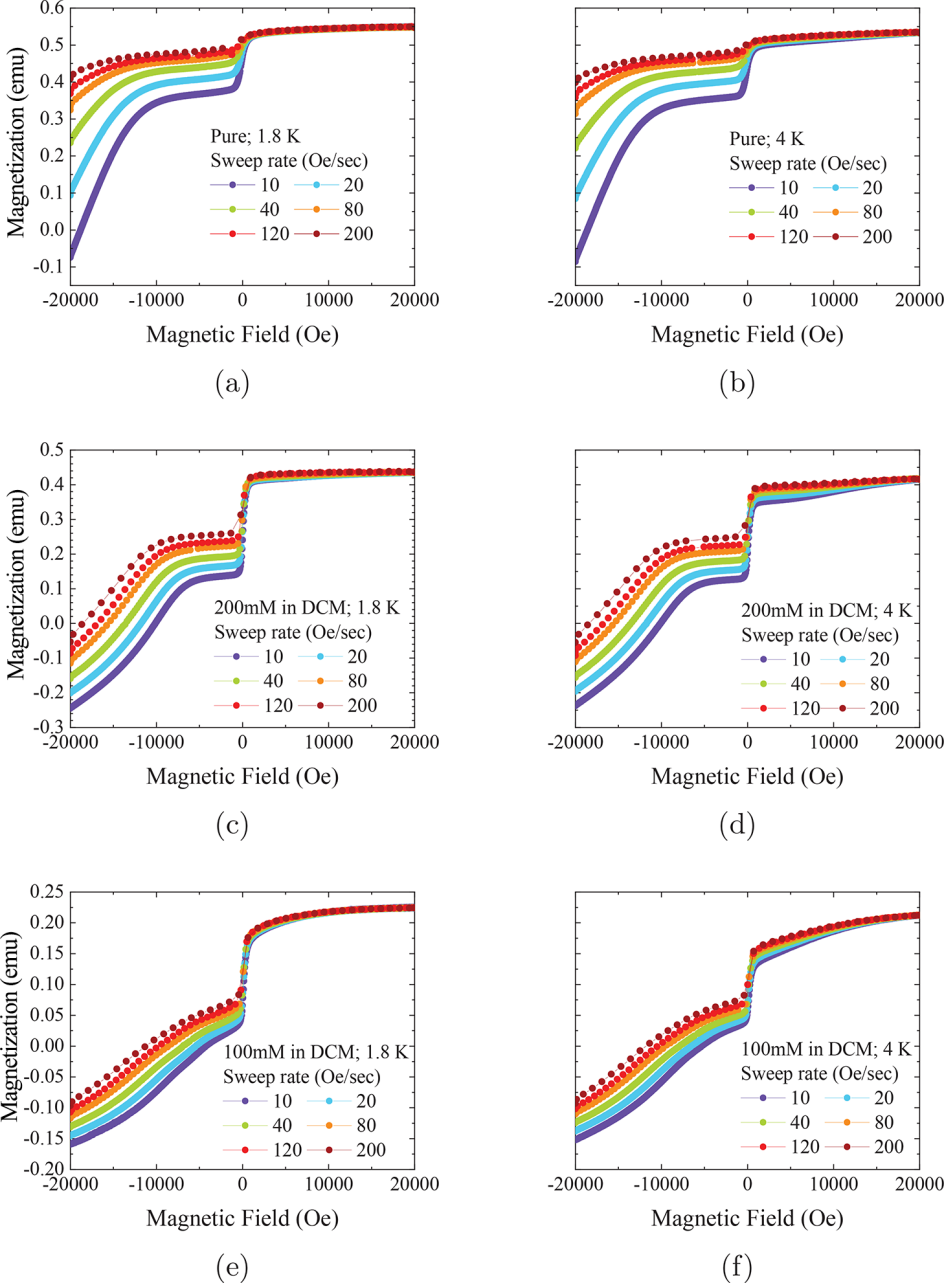
Demagnetization measurements for [Dy(Cp^ttt^)_2_][B(C_6_F_5_)_4_] in DCM and as a pure crystalline solid. Note different absolute values of the magnetization in emu are due to different sample masses, volumes, and concentrations; the absolute values are not important for determining QTM rates. *y*-Axes are shared on horizontally adjacent plots. Measurements for the pure sample were performed using VSM mode, and for the DCM samples using DC mode. Lines are a guide for the eye.

**Figure 4:**
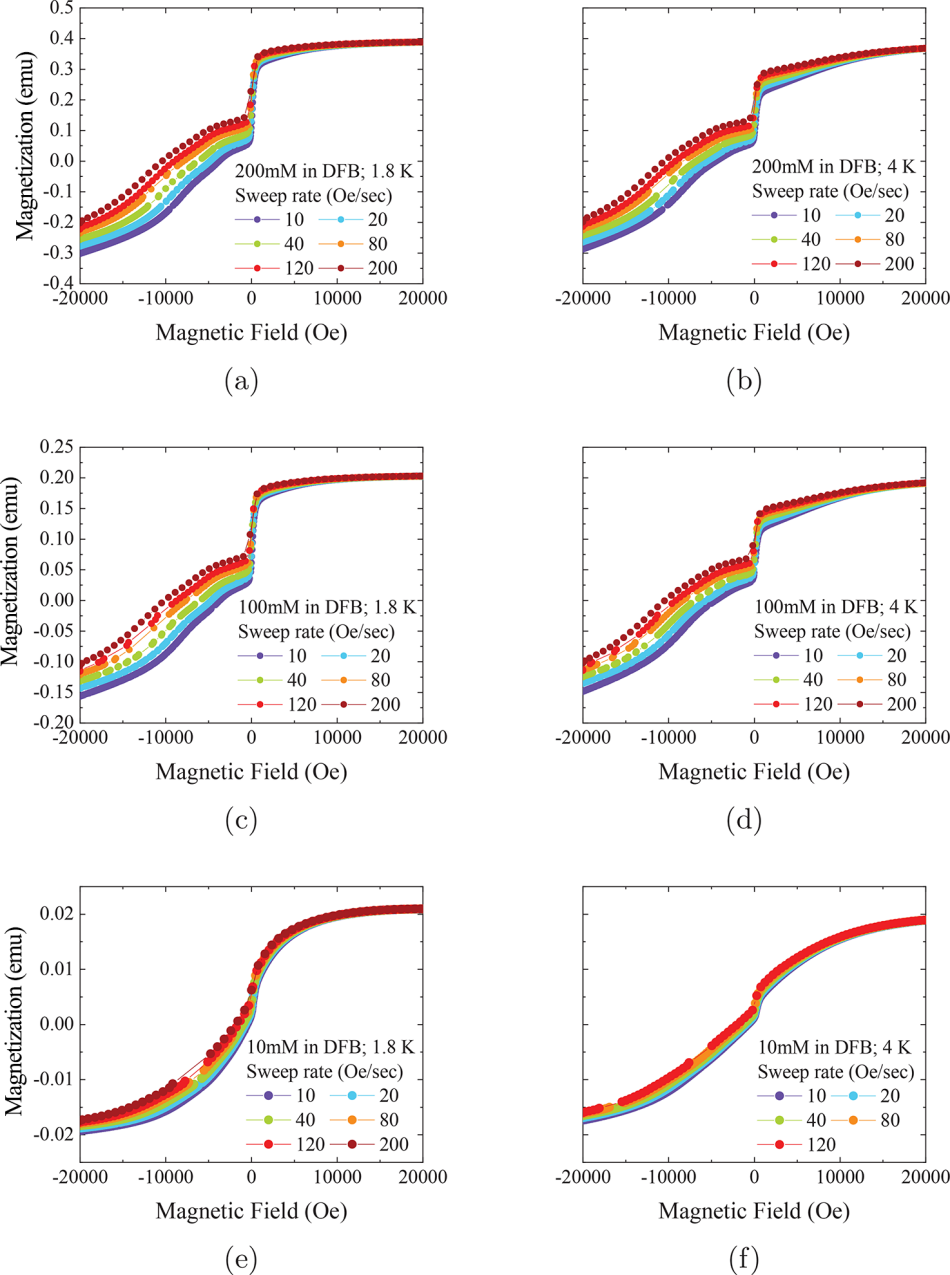
Demagnetization measurements for [Dy(Cp^ttt^)_2_][B(C_6_F_5_)_4_] in DFB. Note different absolute values of the magnetization in emu are due to different sample masses, volumes and concentrations; the absolute values are not important for determining QTM rates. *y*-Axes are shared on horizontally adjacent plots. All measurements were made in VSM mode. Lines are a guide for the eye.

Examining a 200 mM frozen solution of [Dy(Cp^ttt^)_2_][B(C_6_F_5_)_4_] in DCM shows the phase and environment have little effect on the behavior of the magnetization above the zero-field step (Figure 3c). However, the drop at zero-field is larger and appears sharper than for the pure crystalline sample, suggesting increased QTM rates; this is consistent with previous measurements on a ca. 170 mM DCM sample.^2^ Furthermore, the broad downturn below the transition is sharper and at smaller negative fields. At 4 K, the magnetization decrease in positive fields is more pronounced for the slower sweep rates (Figure 3d), but otherwise the increased temperature has little effect. Reducing the concentration to 100 mM results in a more rounded profile in positive fields as it approaches the QTM transition, and the broad feature at negative fields is much shallower compared to the 200 mM sample (Figure 3e). At 4 K, the approach to the QTM transition becomes more linear and the gradient increases (Figure 3f); this suggests that magnetic reversal in this regime is faster than for the 200 mM concentration sample and has more temperature dependence.

Changing the solvent from DCM to DFB appears to have little effect on the features observed in the magnetization sweeps for concentrations of 200 and 100 mM (Figure 4). However, the 10 mM sample in DFB shows a very broad and curved profile on both sides of the transition (Figure 4e, f), suggesting faster magnetic reversal than the higher concentrations; the same characteristics were observed for ca. 20 mM and 40 mM concentrations in DCM and DFB, respectively, in the original work.^2^ We note that it is difficult to accurately determine the linear regimes to obtain *P*_*m*,*m*′_ due to this curvature. This is further exacerbated by the small magnetic moment of these samples leading to a loss of data points around *M*(*H*) = 0.

The data were fitted as described earlier to obtain *P*_*m*,*m*′_ for all samples. For the 200 and 100 mM concentrations, there is a slow decrease in the proportion of spin flips as the sweep rate increases for both temperatures (Figure 5a and b), but there is little dependence on the nature of the solvent (DCM or DFB). The 10 mM in DFB and pure samples have a much smaller proportion of spin flips throughout the entire range of sweep rates, with the former showing negligible change and the latter decreasing as the sweep rate increases.

**Figure 5:**
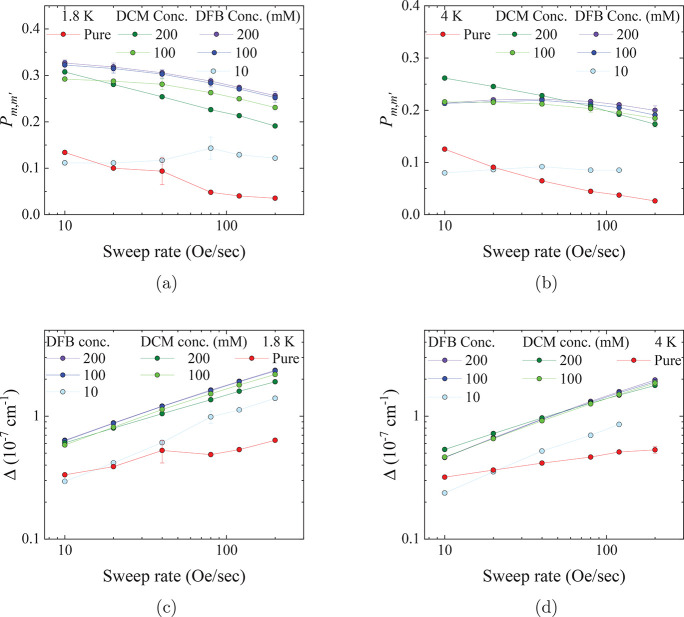
Proportion of spins that reverse magnetization at (a) 1.8 K and (b) 4 K, and ensuing size of the tunneling gap for [Dy(Cp^ttt^)_2_][B(C_6_F_5_)_4_]. Lines are a guide to the eye.

The magnitude of the tunnel splittings were subsequently calculated from these values of *P*_*m*,*m*′_ using eq 5 (Figure 5c and d). Our data show a slowly increasing tunneling gap between 0.6 and 1.1 × 10^–7^ cm^–1^ as the sweep rate increases for 100 and 200 mM concentrations in DFB and DCM. While the tunneling gap is not, itself, affected by the sweep rate, this apparent dependence arises due to the hole-digging mechanism.^24^ The tunneling gap is noticeably smaller by about a factor of 2 for the pure sample, lying between 0.3 and 0.7 × 10^–7^ cm^–1^ in this regime. The 10 mM in DFB tunneling gap follows the same trend as the higher concentrations but is more similar in magnitude to the pure crystalline sample.

As QTM is permitted in Kramers spin systems by nonzero residual transverse magnetic fields, QTM rates are intrinsically linked to the strength of the local dipolar magnetic field, which in turn is dictated by the distance between neighboring magnetic moments. For the pure crystalline sample, the nearest neighbor Dy···Dy distance is 10.4 Å.^2^ For the solution samples, we can approximate the intermolecular Dy···Dy distance by using the Wigner–Seitz radius expression^25^ where *n* = *N*_A_*C* is the molecular density per cubic meter (Table 1); similar results are obtained using the Chandrasekhar formula.^26^

**Table 1:** Average Nearest-Neighbour Dy···Dy Distances for Different Concentrations of [Dy(Cp^ttt^)_2_][B(C_6_F_5_)_4_], as well as in the Pure Crystalline Sample

sample	*d* (Å)	*d*^–3^ (Å^–3^)
200 mM	25.1	6.3 × 10^–5^
100 mM	31.6	3.2 × 10^–5^
10 mM	68.2	3.2 × 10^–6^
crystalline	10.4	8.9 × 10^–4^

The other factor determining the dipolar coupling between SMMs is their magnetic moment. This can be inferred by measuring the magnetization of the sample *M* when approaching the zero field region *H* → 0 right before QTM takes place, which is extracted via a linear fit of *M*(*H*) as detailed above.

For a sample of known volume *V* and Dy concentration *C*, we obtain an average magnetic moment per Dy atom of ⟨*m*_∥_⟩ = *M*/(*CV*) along the direction of the applied field **H**. However, since SMMs in a frozen solution are randomly oriented, their magnetic moment has a nonvanishing component *m*_⊥_ perpendicular to **H**. This transverse magnetic moment can take any orientation in the plane perpendicular to **H**, therefore ⟨*m*_⊥_⟩ = 0 and the macroscopic magnetization is parallel to the applied field. Nevertheless, *m*_⊥_ still contributes to local dipolar fields in the vicinity of a SMM, whose intensity depends on . The relation between the measured magnetization per SMM and the microscopic dipole moment is provided by the orientational (ensemble) average
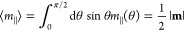
8where *m*_∥_(θ) = |**m**|cos θ and θ is the angle between the SMM easy axis and the applied magnetic field **H**. Microscopic dipolar magnetic fields can then be estimated as
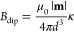
9where κ is an angular factor depending on the relative orientation of **m** and the vector joining two Dy atoms, taking values between 1 and 2 and averaging to 1.38. Although the dipolar field in the polycrystalline sample can be calculated from the crystal structure, a similar reasoning still needs to be applied to deal with the presence of multiple randomly oriented grains. The dipolar field in the pure crystal is calculated by summing the fields produced by all the Dy atoms within a distance *R*_cutoff_ = 300 Å from a reference Dy atom. As shown in Figure 6a, the estimated dipolar fields have similar values across different solution samples, with the exception of the 10 mM solution, as expected due to the much larger average Dy···Dy distance. In the case of a pure crystalline sample, we can also determine the direction of the field. From Figure 6b we infer that the largest component of the field (88%) is perpendicular to the SMM easy axis, and is thus compatible with the presence of QTM.

**Figure 6:**
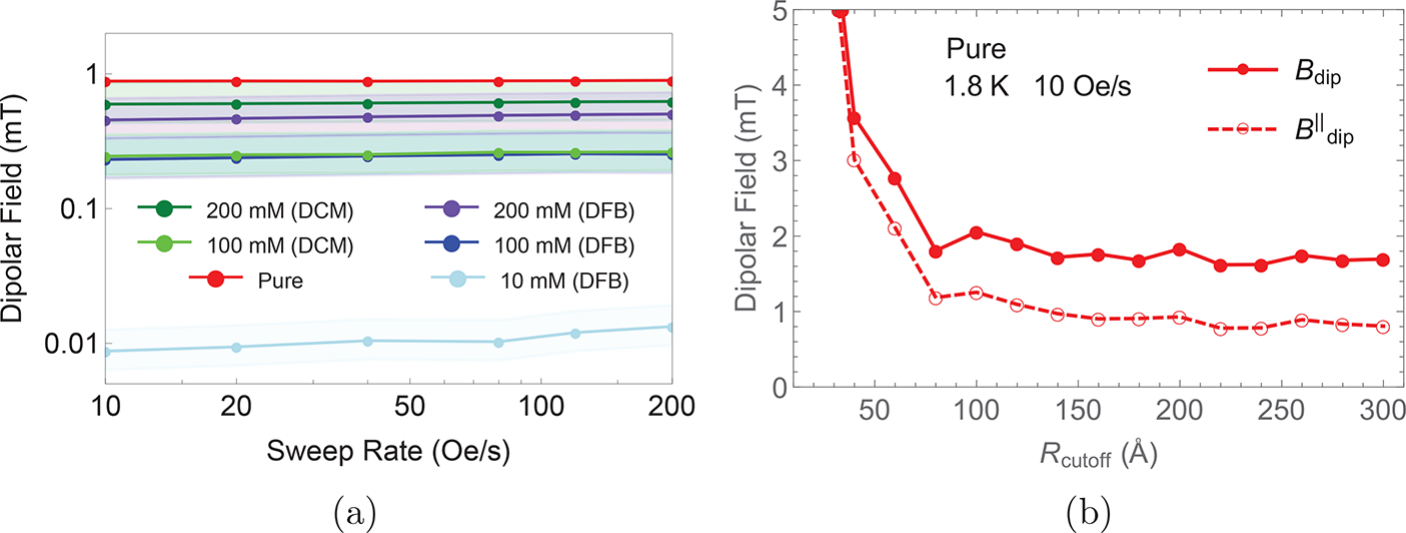
(a) Calculated dipolar fields corresponding to the samples measured in Figure 5 (same color coding). Shaded areas indicate the range of fields obtained for different angular factors κ, whereas dots indicate the orientational average. (b) Dipolar field at a Dy atom in a pure crystal generated by all Dy atoms within a distance *R*_cutoff_. The magnitude of the field is shown as full circles, while the component parallel to the easy axis *B*_dip_^∥^ = **B**_dip_·**m**/|**m**| is shown as empty circles. The magnitude |**m**| was extracted from the magnetization measured at 1.8 K with sweep rate 10 Oe/s.

These results clearly demonstrate that changing between DCM and DFB has little effect on QTM, confirming previous conclusions of Goodwin et al.^2^ They also demonstrate that reducing the concentration of paramagnetic centers increases the average Dy···Dy distance, thus reducing the local dipolar field (the magnitude of of which is proportional to the inverse cube of distance, Table 1) and hence reducing the tunneling gap. However, the pure sample has a similar magnitude of the dipolar field to the highly concentrated samples in solution, yet the tunneling gap is smaller. This suggests that there must be a significant structural and/or vibrational perturbation in solution that increases the tunneling gap for the solvated samples.

Comparing to other SMMs whose tunneling gaps have been measured, we note that the magnitude of the gap in [Dy(Cp^ttt^)_2_][B(C_6_F_5_)_4_] is an order of magnitude smaller than TbPc_2_ (Table 2).^28^ This is to be expected as TbPc_2_ is a non-Kramers ion and as such the zero-field gap is directly influenced by the nonaxial crystal field. The tunneling gap for [Dy(Cp^ttt^)_2_][B(C_6_F_5_)_4_] appears to be on the same order of magnitude as magnetically dilute Li_2_(Li_0.994_Fe_0.006_)N,^27^ and the non-Kramers molecular Fe_8_.^8,21^ The former is a Kramers system, like [Dy(Cp^ttt^)_2_][B(C_6_F_5_)_4_], with no first-order splitting from crystal fields and as such the size of the gap is expected to be similar. The latter, Fe_8_, is non-Kramers and so one might expect an increased tunneling gap; however, the ground state in this compound arises due to magnetic exchange between the Fe ions, and thus its multisite delocalized nature could garner some protection from QTM and thus a decrease in the tunneling gap.^29^

**Table 2:** Tunnel Splittings for a Variety of Other SMMs

compound	Δ (cm^–1^)
Fe_8_^8,21^	≈1 × 10^–7^
Li_2_(Li_0.994_Fe_0.006_)N^27^	≤8 × 10^–7^
TbPc_2_^28^	1.7 × 10^–6^
[Dy(Cp^ttt^)_2_][B(C_6_F_5_)_4_]^2^	0.2 → 1.1 × 10^–7^

Magnetization sweeps for various different concentrations of [Dy(Cp^ttt^)_2_][B(C_6_F_5_)_4_] in DFB and DCM were compared to a polycrystalline sample of [Dy(Cp^ttt^)_2_][B(C_6_F_5_)_4_]. We find that the solvated samples show an increase in the size of the zero-field tunneling gap, even accounting for relative dipolar fields, which we suggest arises from a structural and/or vibrational perturbation to the molecular structure.

## Experimental Details

### Sample Preparation

A pure sample of [Dy(Cp^ttt^)_2_][B(C_6_F_5_)_4_] was prepared using methodology described previously.^23^ A 27.5 mg crystalline sample was pulverized in a mortar and pestle to a microcrystalline powder and secured inside a 5 mm flame-sealed NMR tube using 20.3 mg of eicosane. DCM and DFB were distilled from CaCl_2_ and stored over 3 Å and 4 Å sieves, respectively.

All solution samples were prepared under an inert argon atmosphere through serial dilution of a 200 mM stock solution of [Dy(Cp^ttt^)_2_][B(C_6_F_5_)_4_] (52.3 mg, 0.04 mmol) in DFB or DCM (200 μL). Owing to sample instability in DCM, the solutions were kept below 0 °C throughout. 100 μL of each solution (DFB: 200, 100, and 10 mM; DCM: 200 and 100 mM) was pipetted into borosilicate NMR tubes. Each solution was then frozen by immersion in liquid nitrogen, the head space evacuated, and the tube flame-sealed to a length of ∼3 cm. We can be confident that the samples remain chemically stable following this protocol (decomposition of this complex is accompanied by a diagnostic color change from yellow to pink (not observed here)), and that the decomposition product shows no hysteresis (cf. the significant remnant magnetization observed here).

### Magnetometry

Sweep-rate-dependent demagnetization measurements were made in a Quantum Design MPMS3 SQUID magnetometer. Samples of a known mass or concentration were prepared in sealed NMR tubes and loaded into a straw that was fixed to a translucent glass-reinforced polycarbonate adaptor attached to a carbon fiber rod. The samples were cooled in zero-field to 1.8 K. The samples were initially magnetized at +4 T for 1 h then at +2 T for 1 h before the field was swept to −2 T at the given rate. Before subsequent sweeps, the sample was first saturated at +4 T for 20 min then +2 T for 10 min before the next sweep commences. After all the sweeps were completed at 1.8 K the sample was warmed in zero-field to 4 K and the previous sequence of commands were repeated. For the DCM samples, the DC measurement mode was used, while other measurements were performed using the vibrating sample magnetometer (VSM) mode.

## Data Availability

Research data for this work can be found at 10.48420/21740855.

## Supporting Information Available

Analysis of the magnetometry data of [Dy(Cp^ttt^)_2_][B(C_6_F_5_)_4_] using the KVG model to obtain the tunneling gap and comparing to the LZS model (PDF)
