# Supplementary material for: Measurement of the Quantum Tunneling Gap in a Dysprosocenium Single-Molecule Magnet
Source: J Phys Chem Lett. 2023 Feb 22;14(8):2193–200. doi: 10.1021/acs.jpclett.3c00034 (PMC9986949; doi:10.1021/acs.jpclett.3c00034)
Supplement: Supplementary file 1 — jz3c00034_si_001.pdf [file jz3c00034_si_001.pdf]

Supplementary Information for Measurement of the Quantum Tunneling Gap in a Dysprosocenium Single-Molecule Magnet

William J. A. Blackmore, Andrea Mattioni, Sophie C. Corner, Peter Evans, Gemma K. Gransbury, David P. Mills,* and Nicholas F. Chilton*

Department of Chemistry, School of Natural Sciences, University of Manchester, Oxford Road, Manchester, M13 9PL, UK

E-mail: david.mills@manchester.ac.uk; nicholas.chilton@manchester.ac.uk

The proportion of spin-flips when sweeping over an avoided energy level crossing is defined in the Kayanuma, Garg and Vijayaraghavan (KGV) model by[1]

$$P_{m,m'} = \frac{1}{2}\left[1 - \exp\left(-\frac{\pi\Delta^2}{\hbar\mu_0 g\mu_\mathrm{B}|m-m'|\mathrm{d}H/\mathrm{d}t}\right)\right]. \tag{S1}$$

For ease we define

$$b = \frac{\pi}{\hbar\mu_0\mu_\mathrm{B}|m-m'|\mathrm{d}H/\mathrm{d}t}. \tag{S2}$$

Using the same method described in the main text, we find the powder averaging for Eq. S1 is

$$P_{m,m'} = \frac{1}{2}\left[1 - \exp\left(\frac{-b\Delta^2}{g_z}\right)\right] - \frac{b\Delta^2}{2g_z}\int_{\frac{-b\Delta^2}{g_z}}^{\infty} t^{-1}\mathrm{e}^{-t}\mathrm{d}t, \tag{S3}$$

where

$$\int_{\frac{-b\Delta^2}{g_z}}^{\infty} t^{-1}\mathrm{e}^{-t}\mathrm{d}t, \tag{S4}$$

is the incomplete gamma function $\Gamma\left(0, \frac{-b\Delta^2}{g_z}\right)$. Using Eq. S3 we model the proportion of spin flips to obtain the tunneling gaps at 1.8 K, which are shown in Fig. S1 and Fig. S2. The two models show similar data, with a small convergence as the sweep rate increases. Either both samples are close to the fast sweep rate limit where these models converge,[1] or the sample exists somewehere in between the two models. The KVG model show similar results to the LZS model and is consistent with our conclusions.

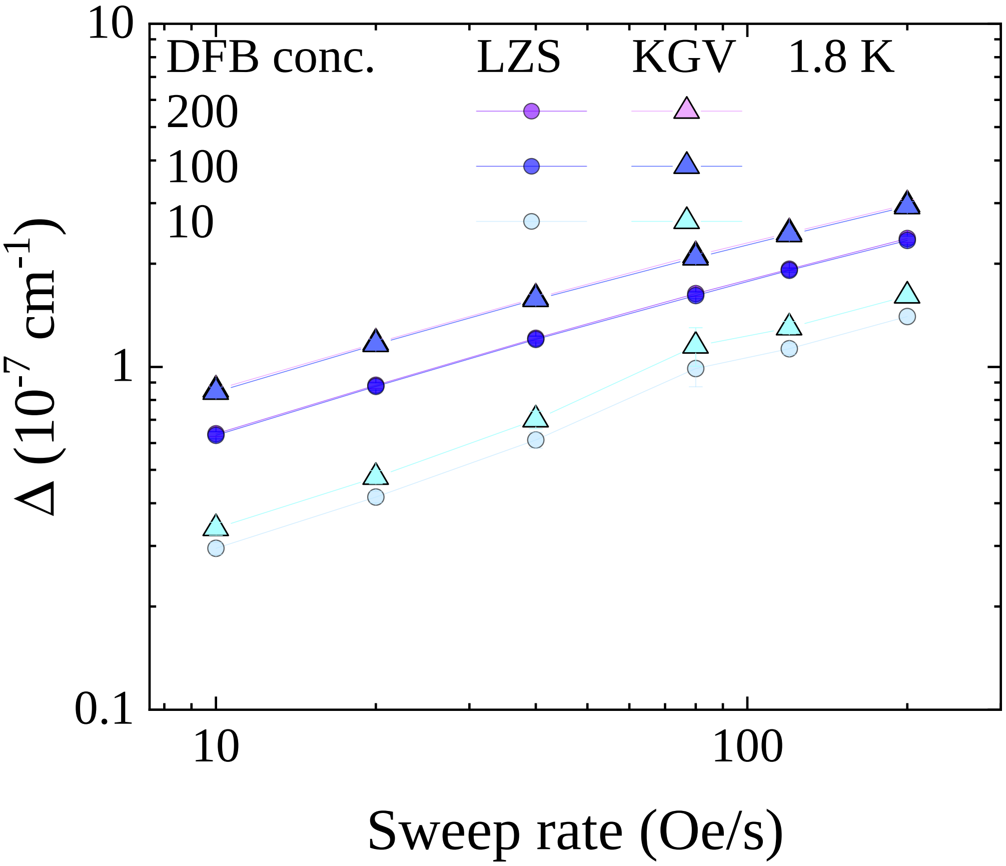

Figure S1: Comparison of the KVG model to the LZS model when calculating the tunneling gap of $[Dy(Cp^{ttt})_2][B(C_6F_5)_4]$ dissolved in DFB. The data shows a small increase in the size of the tunneling gap for the KVG model which converges with the size of Δ for the LZS model at high sweep rates.

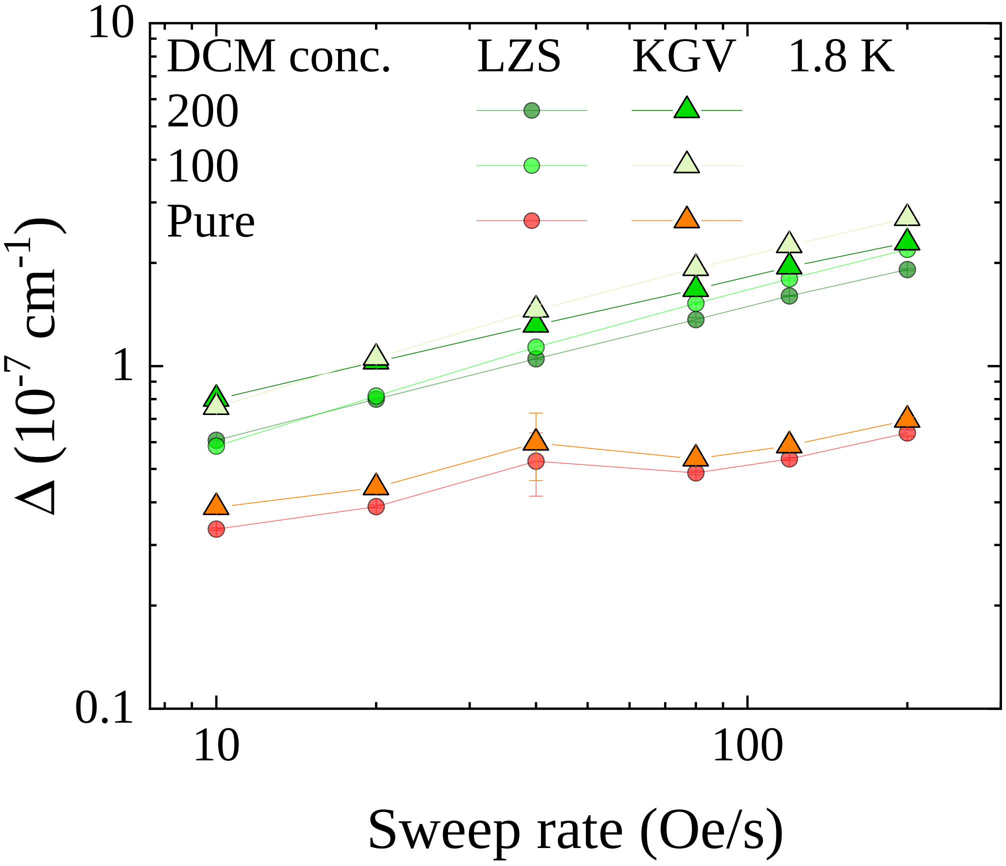

Figure S2: Comparison of the KVG model to the LZS model when calculating the tunneling gap of pure $[Dy(Cp^{ttt})_2][B(C_6F_5)_4]$ and $[Dy(Cp^{ttt})_2][B(C_6F_5)_4]$ dissolved in DCM. The data shows a small increase in the size of the tunneling gap for the KVG model which converges with the size of Δ for the LZS model at high sweep rates.

References

(1) Vijayaraghavan, A.; Garg, A. Incoherent Landau-Zener-Stückelberg transitions in single-molecule magnets. *Phys Rev B* **2009**, *79*, 104423.
